# Being in two minds: accommodating emotional victim narratives in Dutch courtrooms

**DOI:** 10.3389/fsoc.2024.1411155

**Published:** 2024-11-18

**Authors:** Alice Kirsten Bosma

**Affiliations:** Netherlands Institute for the Study of Crime and Law Enforcement, Amsterdam, Netherlands

**Keywords:** observation, victim impact statement, empathy, emotion work, emotional narratives

## Abstract

When Victim Impact Statements (VISs) were introduced in Dutch criminal law in 2005, victims were required to limit their statement to the impact of the harm done by the crime. In 2016, a major amendment lifted this restriction. Even though the statement may (still) not be used as legal evidence, critics worried that the change in scope would invite heightened levels of emotion into the courtroom, which would in turn undermine magistrates’ objectivity. A comprehensive evaluation of the old/restricted legislation and a follow-up analysis of courtroom observations showed that the Dutch system was rather well-equipped to accommodate the expressive function of the VIS before 2016. These studies pay some attention to emotional labor to show how emotional narratives were being dealt with in the courtroom. Recently, a new evaluation of the VIS (post-2016) has been carried out. Observation data of this recent study is qualitatively analyzed and compared to previous findings. The paper also gives insight in the way magistrates manage emotionality in the courtroom in relation to perceptions of objective decision making. Results show that, despite the fact that balancing emotion work with safeguarding objectivity introduces feelings of uncertainty, magistrates accommodate empathy between themselves and the victim, but also open up a space for empathy between the defendant and the victim.

## 1 Introduction

The evolution of victims’ rights, Dutch and global developments alike, has been characterized by an increasing emphasis on the victims’ agency (Bosma et al., 2021; Pemberton and Bosma, 2024). The victim impact statement (VIS), which requires the victim to actively narrate their victimization experience and its aftermath in court, is now often presented as the pinnacle of victims’ rights—the primary vehicle to accommodate victims’ voice (Bandes, 2022). Yet, criticism regarding the fit between emotionally laden stories and the (so-called) objective nature of criminal legal procedure dominates the debate about introducing new and furthering existing victims’ rights. While the VIS is deemed the prime exemplar for finding closure for victims, it is also the most problematic in the range of victims’ rights in terms of magistrates’ emotional labor. In this paper, I analyse observations of cases in which victims presented a VIS to see how magistrates balance victim acknowledgment on the one hand and uphold legal objectivity as a core value of judicial decision-making on the other hand.

### 1.1 The grand narrative of the courtroom: objectivity

In this paper, I hope to show why magistrates behave the way they do and what their motives are in cases where a victim impact statement is presented. To that end, it is helpful to explicate the so-called grand narrative of the criminal justice setting. A grand narrative is the meta-narrative that seeks to place existing practices in a position of progress toward or regress from the originating principle or ultimate end (Bernstein, 1991, p. 102). Put simply, a grand narrative is the ‘story’ that explains, rationalizes and legitimizes the state of affairs (Mäkelä and Björninen, 2022). Even though grand narratives are subject to change and may vary widely over time and in different cultures, grand narratives may be seen as pervasive scripts.

The current persistent script of modern western ideals about the rule of law and legal decision-making favor objective reasoning over emotional influence, and foreground the notion of (positivist) objectivity (Bergman Blix and Wettergren, 2018; Maroney, 2011). The purpose of the court is to decide in matters of conflict in an objective, unbiased, efficient, depersonalized and dispassionate way so that the conflict can be ended. This is illustrated by the appealing image of lady justice. Lady justice weighs matters on an objective scale, while blind to bias and personal distractions.

The traditional conception of objectivity is the direct opposite of subjectivity in the sense of bias and inclusion of personal standpoints and is achieved through typification and standardization (Rogers and Erez, 1999). Indeed, the law aims to structure social relations in forms of claims and counter-claims under established rules that are just “there” (Shklar, 1964). Remaining objective is aided by the internalization of certain legal fictions (Pemberton and Bosma, 2024). Generalizations—that are not necessarily true in the real world—standardize attitudes that promote objectivity. The presumption of innocence would be the clearest example: criminal justice authorities behave as if the defendant is in fact innocent, as not to be biased or drawn in the trap of tunnel vision.

Within the grand narrative of the law, magistrates and other legal professionals embody the “anonymous civil servant” (Dijkstra, 2017). Occupational norms require magistrates to suppress personal feelings, show courtesy and patience and foster remorse (Field and Tata, 2023; van Oorschot et al., 2017), shame and guilt in the offender while discouraging anger, contempt and indignation. Especially when dealing with legal representatives, who are familiar using the highly complex legal language, magistrates seem to construe an air of impartiality and neutrality by ruling out perceptions of subjective involvement. As Roach Anleu and Mack (2005) describe it, the judge “responds to the legal argument, not to a citizen’s particular demands or desires” (p. 591).

### 1.2 Opening up: creating a space for empathy

The above relates to what legal professionals would defend is objectivity. However, the reality diverts from the grand narrative. *Doing* objectivity work in practice has become a matter of balancing engagement and detachment (Bergman Blix and Wettergren, 2018). Lady justice is still blind in current day practice, but rather than not empathizing with anyone, she empathizes with *everyone*. Empathy is used to understand what is at stake for litigants (Bandes, 2009). Those who still frame empathy as a threat against objectivity may have conflated the term with sympathy. Törnqvist (2022) distinguishes the two in a very clear manner: “Empathy implies a knowing of the other person’s experience but does not necessarily include taking any stance on, or caring for, that person or his or her emotions. In sympathy, care is central and we experience someone else’s plight as mattering categorically because we experience that person as mattering.” (p. 266).

The shift toward acknowledging emotions’ place in the courtroom gains more support in legal scholarship and practice. Emotions slowly came to be seen as being not only inevitable, but also legitimate and helpful in judging (Bandes et al., 2021). It is exactly the capacity to judge as an emotional being with a *rechtsgefühl* that makes a human judge to be preferred over an automated machine (Schnädelbach, 2018). Managing emotions in relation to judging can be referred to as emotional labor (Hochschild, 2003 [2012]). Emotional labor is performed both to manage other subjects’ emotions and own emotion, with the goal of producing a proper state of mind.

### 1.3 Emotional narratives: victim impact statements

The narration of victimological experiences presents a crucial challenge to the grand narrative of objectivity that the law tries to uphold (Pemberton and Bosma, 2024; Shklar, 1990). Previous research has demonstrated that magistrates’ attitudes change in the presence of the victim (Haket, 2007), which is often evaluated as a threat to objectivity. However, the re-emotionalization of law (Karstedt, 2011) gradually created more space for interaction with victims and their stories, such as via victim impact statements.

Research on victim acknowledgment stresses the importance of legal personnel’s empathy. Victims’ likelihood of deciding to continue to engage with the legal process as well as their overall satisfaction or perception of procedural justice has been linked to the level of empathy that victims experience throughout the criminal justice system (Goodrum, 2013; Rudolfsson, 2022). It is certainly true that empathic feedback is necessary for victim impact statements to reach their desired outcome, to account for their acknowledgment and possibly even to help the victim in their way toward healing (Bandes, 2022).^1^ Proponents of the VIS have emphasized that not only the victim benefits from acknowledgment, but that the educational value of the VIS, as it might elicit empathy and even remorse on the defendant’s part, also benefits the defendant and society as a whole (Bibas and Bierschbach, 2004).

This does not mean that now that emotions are more widely recognized as deserving a place within the law, judges, as if magically, know how to balance the pre-requisites to be both empathic and respectful toward victims and remain objective in decision making (Rudolfsson, 2022). It is well documented that professionals find working with victims’ emotions both rewarding and demanding (Roach Anleu and Mack, 2005; Rudolfsson, 2022; Shuler and Sypher, 2000). Emotionally intense interaction is something that is not included the training of many judges, and does not become habitual in the everyday experience of magistrates, as the number of VISs that are presented are relatively low in comparison to the number of cases that they judge.^2^

### 1.4 The Dutch victim impact statement

In this paper, I study how magistrates manage courtroom interaction in relation to the victim impact statements (VIS) in my home country, the Netherlands. This requires some background information about the Dutch criminal proceedings and the legal implementation of the VIS in particular.

Dutch criminal proceedings adopt a largely inquisitorial approach. Victims can exercise certain rights in the capacity of ‘*procesdeelnemer*’—which roughly translates as ‘participant to the proceedings’. Victims only have legal standing as a party in their capacity of civil claimant (art. 51f CCP). In contrast to adversarial proceedings, the judge not only manages the proceedings, but also has an active role in scrutinizing the evidence. It should be noted that evidence is laid down in the written case files, the *dossier*, which have been very carefully compiled by the prosecution before the start of the trial, and made available to all parties. The result is that interrogation of witnesses and victims in court is very rare. Rather, their statements have been taken *pre-trial* by the police or investigative judge, and the transcripts are in the dossier. The large majority of trials take place in the absence of the victim.

Criminal legal proceedings are not bifurcated. The hearings include the presentation of the indictment, the interrogation of the defendant, witnesses* and experts*, the overview of the case files, the presentation of the VIS*, the prosecutor’s address, clarification of the civil claim*, the statement of the defense, counter-pleas of the public prosecutor and defense, and the defendant’s last word.^3^ The placing of the VIS has a performative function to elicit reactions from judge, prosecutor and defendant.

In terms of courtroom configuration, it should be noted that the victim is usually located with the audience, in the front row of the public gallery. This gallery is, in most cases, not fenced off from the central performance zone. The defendant faces the judges (right in front) and public prosecutor (desk slightly ajar from the judges’ bench on their right).

The Dutch criminal code of procedure allows everyone qualifying as a victim to submit a written victim statement (art. 51b CCP) to the public prosecutor and request to add it to the case files, while only victims of crimes that carry a maximum sentence of 8 years of imprisonment or higher, as well as some crimes specifically named by the legislator, can deliver an oral statement in court (art. 51e CCP). Until recently, only oral-VIS-eligible victims were explicitly invited to present a statement (in writing, orally or both—the statements need not be the same). For that reason, hereinafter, when referring to the VIS, I refer to both modalities, but will describe the legal requirements of the oral VIS.

Eligible victims must be aged at least 12 years, younger children may be represented by their parents. Surviving relatives of the deceased victim qualify as victims according to art. 51a CCP, and thus do not represent the primary victim but deliver their VIS in personal capacity. For purposes of manageability, the legislator has limited the number of oral presentations in court to a maximum of 3. The victim may assign a representative to deliver the VIS. In practice, if the victim assigns a representative, this is often a victim support worker or the victim’s lawyer. These representatives often help drafting a written version of the VIS and prepare the victim for the presentation thereof. As there are no reliable statistics about the number of VISs, there is no reliable information about the prevalence of VISs read by representatives either. My general experience is that if victims opt for the oral version of the VIS, they are encouraged to present the VIS themselves.

The VIS is a relatively new concept in the Netherlands, first introduced in the code of criminal procedure in 2005. Since its introduction in 2005 and evaluation in 2010, some major amendments have been implemented, most notably regarding the scope of the VIS.^4^ Before 2016, victims were only allowed to include the impact of the victimization on their life in their statement. Opinions on evidence, guilt, the desired sentence and other remarks were not allowed, as they were thought to interfere with the presumption of innocence and pose a risk for the judges’ impartiality. Despite minimal interruptions when victims tended to go beyond the allowed scope (Lens et al., 2010), many victims experienced the scope as impeding their wish to freely recount their experience of victimization and its aftermath. In 2016, the VIS became unrestricted in its scope. What has not changed is the influence that the VIS may have on legal decision making: it cannot be used as evidence, and may only “accentuate” the decisions that were taken on the basis of the rest of the casefiles.

The implementation was first evaluated in 2010 (Lens et al., 2010). Lens and colleagues found that the VIS meets a clear need for victims of crime. Especially victims of the more severe crimes appreciated the possibility to submit a VIS. The choice of modality was a matter of personal preference. Victims who were concerned they might not be able to control their emotions in court choose to submit a written VIS, while victims who stressed the importance of being able to voice their own opinion in court used the oral VIS. Communication with the offender and the judicial authorities was the main reason for participation. Although the law at the time did not allow for it, influencing the outcome was also important for the victims interviewed. Submitting an oral or written VIS turned out to have a small positive effect on the perceived control over emotional recovery and the experience of procedural justice. Delivering a VIS did not diminish the victims’ anxiety or their anger toward the offender.

Data from the same study was used to study the expressive function of the VIS (Booth et al., 2018). Booth and colleagues found that the Dutch approach to the VIS was characterized by a rather flexible approach, that allows for a greater scope for victims to tell their story compared to implementations of the VIS in adversarial countries. On several occasions, victims were provided with multiple opportunities to tell their story. In terms of being heard, the study found that magistrates (especially prosecutors) directly acknowledged the content of the VIS and provided defendants with an opportunity to directly respond to the victim’s statement, thereby facilitating important occasions for demonstrations of remorse and further acknowledgment from the defendant.

A second evaluation of the VIS—with its new scope—has been carried out only recently (Kragting et al., 2022), 6 years after the extension. This evaluation does not directly investigate emotional labor in the courtroom. In between the two evaluations of the VIS (post 2016), I carried out an experimental study with magistrates responding to video vignettes of victims delivering a VIS (Bosma, 2019). Data suggested that magistrates were performing emotional labor in response to the VIS. They were actively creating a calming atmosphere in the courtroom so that the victim would sufficiently be at ease to deliver the VIS, while at the same time suppressing own emotional expressions:

“And sometimes that is a little… you would like to, as a person, you would like to say more. But you should stay in your role as a judge and you should protect your impartiality. And thus, you cannot say too much. I needed to get used to that again.” (p. 157).

Furthermore, results from the same study suggests that magistrates struggled to find the right empathic balance, especially if the victims’ narrative is a sad one. They explained that they would empathize more with the victim than they, beforehand, were prepared to, because they would “go through it with” the victim (p. 163).

## 2 Materials and methods

My current analysis is based on observation research that was carried out as part of the VIS evaluation study in 2022 (Kragting et al., 2022). From November 2021 to June 2022, 25 criminal legal hearings where victims were supposed to present an oral VIS were attended.^5^ In two cases, the victim did not present a VIS, but in the 23 cases which did include the presentation of an oral VISs, 42 VISs were presented. Nine victims were related to the defendant (family *n* = 4; acquaintance such as neighbor, colleague *n* = 5). Time that had passed between victimization and court hearing varied widely: median: 18 months, ranging from 24 days to 40 years.^6^

Observations were geographically spread across the Netherlands (7 out of 10 judicial districts) and across the two types of divisions that deal with VIS-eligible cases: the single-judge criminal division (*n* = 10) and the three-judge criminal division (*n* = 13).^7^ Crimes included homicide, arson, aggravated assault, sexual crimes, deprivation of liberty, threat of homicide, stalking, robbery, war crimes,^8^ and traffic offences.

Observers made notes on the following themes. First, the way the presiding judge offered the opportunity for presenting the VIS (e.g., words used to give the floor to the victim, instructions on where to stand, etc.). Second, the VIS itself: observers noted to whom the VIS was addressed (e.g., the court, the defendant, society in general, the primary deceased victim or different), which topics the VIS addressed (such as the [emotional] impact, the crime itself, criminal evidence, culpability of the defendant, desired punishment, procedural justice). Third, observers attended to verbal and non-verbal responses to the VIS from the judge(s), the public prosecutor, and the defendant. Last, observers took notes on the victims’ emotional display before, during and after presenting the VIS.

I analyzed the notes from the observers qualitatively, looking for cues that indicated that magistrates performed emotional labor. This means that I looked for notes on emotional expressions by any of the parties (including the victim), on the perceived atmosphere in court, and particularly for notes that signaled a potential change in atmosphere or emotional expression. For example, notes on judges offering a glass of water or a ‘way out’ were helpful indicating emotional labor, as were notes on the public prosecutor repeating victims’ words. Gestures and other non-verbal information were also taken into account. Emotional expressions could easily be deducted from non-verbal behavior: e.g., shame from averting gazes, speaking very softly and making oneself as small as possible. I analyzed spaces of empathy by coding notes that were indicative of active listening, dialogue, growing mutual understanding, and affirmative communication. I structured my findings in parallel to the chronology of the criminal hearing.

## 3 Results

### 3.1 Preparations

Dutch legal proceedings are characterized by a strong emphasis on pre-trial investigations. The trial hearing is thus a carefully pre-planned ritual that leaves little room for spontaneous interruptions. Oral presentation of statements is limited compared to common law practice. Like the rest of the trial, victim participation is also carefully pre-planned. The victims’ first contact with the judiciary is when the public prosecution service registers the case and sends out a registration form with inquiries about the victims’ wishes: does the victim want information, to claim damages, to deliver a written and/or oral statement? If the victim does not return the form,^9^ they will not receive additional information about the course of the proceedings.

In general, all communication up until the trial between the judiciary and the victim is in writing, and is hardly to be called a ‘dialogue’. As Ryan (2023) explains, bureaucratic forms shape what is registered and how the personal identity—of, in this case, the victim—is documented in the eyes of the state. Whether the victim is registered as an active participant depends on the form. This is only different for victims whose case is tried by a three-judge criminal division and who are eligible to perform a VIS: they are invited (again, through the same form) to meet the public prosecutor prior to the hearing. This meeting is used to prepare the victim for the trial and to introduce them to the court house. In practice, only a very small minority of victims make use of this opportunity. For victims who do not meet the prosecutor prior to the hearing, the hearing is often their first introduction to the court, which may be quite a daunting experience in itself. The preparation of the VIS itself is up to the victim. For some, it is always in the back of their minds, because they are unsure how to deliver the VIS (Kragting et al., 2024).

### 3.2 Announcement of the VIS

The presiding judge opens the hearing and hands the floor to the different speakers. All parties communicate via the presiding judge. At the beginning, the presiding judge opens the hearing, often welcoming all parties individually, also tending to the victim. It happens that the judge seeks confirmation about the victims’ intention to present the VIS at the beginning of the hearing, but that depends on the circumstances. After the indictment is presented by the prosecutor, the defendant is interrogated by judges and prosecutors, and the judge has given an overview of the case files, the floor is handed to the victim for the presentation of the victim impact statement. Up until that moment, there interaction with the victim is virtually absent in most cases.

When the floor is handed to the victim, the victim is not always sure what to do: stay at their place in the public gallery or move around, speak freely or read a written VIS aloud. They might even leave the presentation to a representative such as a victim support worker. The observation data does not show that the presence of a victim support worker or victim’s lawyer alleviates the victim from this uncertainty. They may have discussed the presentation of the VIS prior to the hearing, but given the choice where to stand and what to do at that exact and conceivable important moment, seems to evoke uncertainty in the victim nonetheless.

The observation data shows that the judges often encouraged victims to at least try to present the VIS themselves, because they had chosen to attend the hearing and had the intention to present a VIS. Judges explicitly left open the possibility to change their minds: if they would feel they could not do it—even if half way or near the end—the judges said they could still hand the presentation over. Judges were actively trying to foster a calm environment in which the victim could present the vis. By normalizing the feeling of being upset, they would communicate that the victim was allowed to show these emotions. However, the following quote from one of the observations also shows awkwardness in face of intense emotions:

*Judge:* “It is conceivable that this hearing will stir emotions, and that is understandable, and this can happen in a VIS. You can express your emotions, but not without limit. It is essential that everyone who speaks can say whatever they want, from their own perspective, and that they feel free to tell their story. If you cannot control your emotions, you may retreat from this courtroom to the main hall of the building. That happens more often, that is not a strange thing to do at all.”

First, by saying that emotions cannot be expressed without limit, it is unclear what the judge means. Relating the quote to my previous research (Bosma, 2019), I would take it to mean that (extreme) anger, especially when expressed in the form of swearing, very firm accusations of guilt or a direct addressal of the defendant (rather than speaking via the presiding judge) would not be allowed. My research showed that judges are much more lenient in allowing expressions of sadness, and—as the current observations also show—generally take their time to let victims finish their story, even if they are overcome with emotions and need to regain their breath before they can continue. To the ear of a nervous victim who has previously never visited a courtroom, however, the statement may cause confusion.

Secondly, and this was noted frequently in other observations as well, the statement shows that judges tend to try calming victims by giving them an ‘escape’ if necessary: leaving the courtroom. Although the main hall of the building—to which most courtrooms give direct access—might indeed be a calmer place in the sense that it is free from discussion about the case and it takes the victim away from the defendant, it should be noted that the hallway is far from calm. It is often a busy place where litigants, lawyers and public move around. It is possible for victims to reserve a private room to withdraw to, but this option is fairly unknown and was never mentioned in the observed trials.

This shows that as soon as the victim is invited to actively participate in the trial, feelings of uncertainty arise. The victim is uncertain how to perform the VIS. Judges do not fully alleviate this, as there are no clear guidelines on how to resolve these uncertainties.

### 3.3 Presentation of the VIS

The observed cases show the presentation of the VIS proceeded in a civil and calm manner; interruptions were rare. In some cases, especially when the defendant had been quite talkative and inclined to interrupt the magistrates during previous discussions of the case files, the judge urged the defendant to listen very carefully, and if necessary, warned them to not interrupt.

“*One more thing:* you just had a lot of time to talk and I have let you finish your story. Now [victim] will talk, and I don’t want you to interrupt her.”

In another case, where the defendant and his lawyer had spoken very accusatory about the victim, the judges had not reprimanded them before the VIS, neither had there been any interaction between victim and judge before the VIS. However, before the judge gave the floor to the victim, the judge addressed the victim and said:

“A lot has been said about you just now. But you are not the defendant. Just say what you want to say. You can just take a seat and talk.”

This statement recognized that the remarks that were made about the victim might have hurt, and that the victim might have experienced uncertainty about the value of the statements made about them. The judge empathized with the victim, without elevating the victims’ plight as mattering categorically (sympathy—see Törnqvist, 2022), because the judge did not stop the defendant from defending their case in the way they choose.

While presenting the VIS, magistrates were often watching the victim or the case files, sometimes taking notes, reading along the earlier submitted written version of the VIS. Prosecutors were focused on taking notes and watching the defendants non-verbal response to the VIS. Although to the observant’s eye magistrates were actively listening, to the victim who might be unfamiliar with the composure of the court, the appearance of the magistrates—especially those looking up, seemingly lost in their thoughts and those reading case files on their computer screens—might have given an impression of indifference.

The defendant, who was seated in front of the victim presenting their VIS from their place in the public gallery, did not often turn around. Rather, defendants often sternly kept their gaze at the judges. This, however, should not necessarily be interpreted as a sign of disregard for the victim. Defendants were instructed to speak via the presiding judge, and thus for that reason may remain their position facing the bench. Moreover, many defendants seemed to be nervous, especially in relation to listening to the VIS, and might be unsure how to react. In one observed case, the judge asked the victim to show their scars. The defendant did not turn to see before the judge asked him explicitly whether he had seen the scar, and was quick to turn back. Again, the way this interaction is valued by inexperienced court-users may highly differ from this interpretation that is informed by knowledge about court habits and procedures.

In the observed cases, victims were not cross-examined as a result of what they presented in their VIS. Cross-examinations at trial are quite rare in the Netherlands. The civil law tradition puts a lot of emphasis on pre-trial investigations, so that most witnesses are interrogated by either police or the investigative judge. Moreover, as the VIS may not be used as evidence in the determination of guilt, there seems to be little reason for cross-examination. However, many people worried that the extension of the VIS in 2016 would put the victim at risk of secondary victimization because VISs that would address the topics of guilt and proof would lead to cross-examination. In one observed case, the defendant’s lawyer used information from the VIS to pose questions about the claim for damages, but the judge did not question the victim about this, although in the announcement of the VIS, the judge said:

“She will tell what it did to her. Everyone will then get the opportunity to ask questions – also to you [victim].”

In another case, the defendant’s lawyer responded to the VIS by requesting to adjourn the session for further investigations, namely the cross-examination of the victim-witness by the judge commissioner. This request was denied.

### 3.4 Defendants’ responses to the VIS

Judges acknowledged the presentation of the VIS mostly by shortly thanking the victim. In most cases, the judge then gave the floor to the defendant to react. The reactions that defendants gave varied widely. The defendants who pled guilty were often quick to take up their responsibility and say sorry. Some clearly came across as ashamed, speaking softly and under their breath, almost inaudible, sitting in their chair, bend forward with sunken shoulders. Even when defendants did not plead guilty, some acknowledged the harm that was done.

“So harmful. I genuinely did not want this to happen.”

The judge did not allow the defendant to directly address the victim, and interrupted the defendant when they tried to do so:

*Judge:* “Please address me. What would you like to say to her?”

*Defendant:* “I feel really sorry. It should never have happened. Sorry for all the harm.”

Other defendants were showing less self-reflection and regard for the victims’ misfortune. In a case of sexual groping, the defendant remarked:

“Well, I can say now: I’m sorry. 16 months have passed since [the crime], and the victim has now recovered from it as well.”

The judge interrupted by saying that might not be entirely true, looking at how impacted the victim is in court. Later, the judge added that the defendants statements about himself needing to recover may sound harsh to the victim. The victim was nodding in response in the background, indicating that she interpreted this as acknowledgment for her harm.

When the defendant remained silent after the presentation of the VIS, some judges tried to encourage the defendant to open up. The VIS gave the judge an opportunity to empathize with the victim themselves, but also to encourage empathy between victim and defendant. Emotion work is ‘triangulated’: calming the victim after the VIS has an impact on the defendant, and calming the defendant has an impact on the experience of the victim in court.

In the following example, the judge was explicitly asking about the feelings of the defendant (rather than questions about evidence and case facts).

*Judge:* “I’m curious what the defendant thinks.”

*Defendant:* “No.”

*Judge: “*How does this make you feel?”

*Defendant:* “I do not want to talk about that. I cannot say anything about that.”

*Judge:* “You clearly feel uncomfortable though. They [the victims] would like a response. That is quite understandable.”

*Defendant:* “Sorry Mrs. Judge (then he looks down).”

When there was no reaction from the defendant in another case, the judge filled in what the victim might have wanted to hear, namely an apology. The following quote again is a reflection of a judge empathizing with the victim—putting themselves in the victims’ position. By explaining that this is how a hearing works, the judge also respects the defendant’s choice not to, but signals that making an apology and showing remorse would be the right thing to do, stressing what would generally be seen as ‘good behavior’ from the defendant (Tata, 1997).

“Yes, that is difficult because you won’t get a ‘sorry’. I understand you will be disappointed, but this is how a hearing works.”

### 3.5 The prosecutorial response to the VIS

The next step in the criminal procedure is for the public prosecutor to give their address. Especially in the lengthier trials, the judge adjourns the hearing to “cool out” (Booth, 2012) before the prosecutorial address. The break gives everyone some time to process the emotions that were triggered by the VIS.

The VIS is positioned in the criminal proceedings before the prosecutorial address because this allows the prosecutor to take the VIS into account. Booth et al. (2018) found the prosecutorial response to be a unique and key indicator of the accommodation of victims’ voice in the Dutch criminal proceedings. The current observations show that the prosecutorial response is still very much present today.

In a Danish study, Johansen et al. (2023) found that police officers, prosecutors, victims’ counsel and judges each interpret victims’ feelings according to their own professional roles and motivations so as to gain an overview of a case and the actions required of them in relation to it. Prosecutors have a role that clearly differs from the judge’s role. In the Dutch system, prosecutors are magistrates and are thus not directly opposite to the defendant, but often their rationale aligns with the victim’s interest. They can therefore (cherry-pickingly) use some of the information as brought forward by the victim (similar findings in Bandes, 2022). The observations showed that prosecutors sometimes quote, and often rephrase, parts of the VIS.

“As we just have heard in the VIS (…)”

The prosecutor may take a more judgmental attitude in relation to the defendant on the basis of the VIS than the judge can.

The public prosecutor said that the defendant had behaved like a “bastard”, as “is evidenced by the VIS that had just been read by the victim.”

The prosecutor thus comes closer to sympathizing with the victim than the judge, but as it is only instrumental, it cannot be argued that the prosecutor places the victims needs as a categorical rule. The prosecutor also made instrumental use of emotions in the observed cases. First, complimenting the way victims had delivered their VIS, thereby fostering relief and feelings of pride in the victim. Second, they promoted fear and guilt in the defendant by explaining the seriousness of the situation. At the same time, this could also have a signaling effect with regard to the seriousness of the case toward the judges, underlining the message of their address.

“I think you are all affected by what [victim] just told”. The public prosecutor repeated the serious consequences that the assault had on the victims’ family life and work life, and recounted that even in delivering the VIS it became clear how much difficulty the victim still has in the act of talking due to the injuries. The public prosecutor quoted one sentence from the VIS in particular: “I want to go back to how it was, but that is no longer possible.”

The first sentence of the second example, which the prosecutor phrased descriptive, was clearly meant prescriptive: people *should be* shocked by the seriousness of the injuries that the victim had sustained. The prosecutor did not say he was angry, but there is a clear indignation about what happened. According to Milka and Lemmings (2017) the magistrates’ anger may act as a proxy of state- and societal anger, and that seems to explain this fragment of the observations. The direct quoting of the victim about the impossibility of returning to the state prior victimization underlines this.

Shock is more often used as to signal a prescriptive state of anger. In one case, the prosecutor discussed camera evidence, and linked it to the VIS in which the victim had told to have watched that CCTV footage.

“From the VIS it was clear that the victim was shocked by the video-evidence. I was also shocked.”

Victims seemed to feel acknowledged by the remarks of the prosecutor. One victim broke into tears when the prosecutor remarked the following, while making eye-contact with the victim on the public gallery.

“I got the chills while reading [about the injuries]”. “I saw emotions in the claimant, the victim was being consoled just now. Still, six years after the incident, it impacts him.”

### 3.6 Managing the defendant’s emotions after the VIS

After the VIS has been delivered and the prosecutor has given his address, the civil claim for damages is discussed. In this part of the proceedings, there were again possibilities for the victim to speak up. But also afterwards, during the statement of the defense, counter-pleas of the public prosecutor and defense, and the defendant’s last word, the victim’s perspective seemed to linger in the discussion. For example, in a case when the defendant explained his actions one more time and said that he “panicked,” the judge promptly responded, saying:

“And so was [the victim], I think. You can imagine quite clearly that when this happens to you, you feel less safe.”

When the defendant in a case of assault that led to very severe injuries told the judge that after the court case, he wanted to say sorry, the judge asked why not right here and now. The defendant asked his lawyer whether this was the right moment, and then turned around:

“I am sorry. I never wanted you to be like this. I regret it a lot. It should have never happened this way.”

Referring to the victims’ need to find answers also proved to be a way for the judge to get the defendant to talk more about their behavior. Because of the VIS, the judge is not only empathizing with the victim, but also increasingly learning about the personal motivations of the defendant. In a case of arson, the defendant was accused of setting the house of his family on fire. One of his sons did not survive the fire. The defendant had a hard time opening up and talking about the case.

*Judge:* “There are co-victims in this room, your kids and your ex-partner. Do not you want to clear some things up, for them?”

*Defendant:* “No.”

*Judge:* “Answers may be important for your family.”

*[…]*

*Judge:* “Would you like to say something to the victims?”

*Defendant:* [remains silent, but nods and cries].

*Judge:* “Might they have the slightest right to? To hear something from you?”

*Defendant:* “I do not know what to say to alleviate the pain. I’m not sure I was aware of it. Of course, I’m so very sorry, but if I say that, it does not feel enough. It feels contradictory even. I find it difficult… I did think about why it happened of course, and how I felt then, and I do not want to use that as an excuse.”

Afterwards, the defendant was much more open and tried to answer the judges’ questions at greater length. However, this strategy did not always turn out successful. In a case of stalking, the defendant starts off showing some regret, stating that his behavior was due to frustration. As the hearing progressed, the defendant seemed to grow frustrated with the procedure and requests to talk more about his behavior. He stated that he does not want to be in one room together with the victim, and said that he was “done looking at the victims’ face,” even though he was not directly facing the victim and did not try to communicate with the victim apart from these complaints.

When defendants got rude or accusatory toward the victim, the judge interfered but not very sharply. An example was already discussed in paragraph 3.2 where the judge showed dismay about the “talk about the victim” prior to the VIS. In one case, where the atmosphere had been quite tense, the defendant consistently referred to the victim in very rude terms, such as “whore” or “prostitute.” He asked the judge whether he could “ask this whore a question,” to which the judge responded that this was not allowed, and that the victim did not have any other role than being the civil claimant. Prosecutors take more room to correct the defendant. The prosecutor said it “triggered” them to hear the defendant’s lawyer state that the victim is to blame. She then turned to the victim to confirm that it was not provocation.

“For the victim, I wanted to state this very explicitly.”

These examples of a judge trying to get the defendant in a talkative mood relate to cases in which the victim and offender were (once) related, they were family or ex-partners. In case victimization is a result of escalated family contact, it seems that judges try harder to establish a space of indirect dialogue between victim and offender. In a case of escalated play-fighting between an uncle and his nephews, the uncle played far too rough with the boys and ended up assaulting their mother. Having heard the victim refer to emotions of fear in the VIS, the judge asked the defendant whether the children would need to feel afraid of him in future encounters. The defendant answered:

“No, they shouldn’t be, but I can understand if they are”.

The judge then proceeded to ask whether the victims (mother and children) were to blame to any extent for what happened, which the defendant explicitly denied. Questions like these do not only an empathic judge, but also allows for mutual empathy between victim and offender.

### 3.7 The verdict

In a three-judge division, the verdict is delivered 2 weeks after the closing of the hearing. Interested parties may attend the delivery of the verdict. This tends to happen only in high-profile cases. In a single judge division, the verdict is delivered right after the hearing is closed, so that parties are still present. In the observed cases, this provided the judge with an opportunity to explain the decision in person. In a case of acquittal, the judge turned to the victim. The judge explained that the acquittal did not mean that they thought the police report that the victim filed was illegitimate. The judge asked the victim whether everything was clear. This does not require the judge to move outside their professional objectivity, because the case was already closed. Even if the defendant or public prosecutor would appeal, another judge would try the case. So, if the case is closed, the judge seems to have more room to interact with the victim. In one of the observed cases, the judge asked the victim:

“How does this all sink in? What did you hope to get out of this hearing? How will you feel when you travel home?

The extra attention that the judge may give to the victim at the end of the trial may enhance victims’ perceptions of procedural justice and legitimacy of the court, because they feel heard.

## 4 Discussion: being in two (or more?) minds, establishing empathy

The above observations show that magistrates may find themselves in two minds when performing accommodating the victims’ voice in the courtroom. Being in two minds first of all referring to the uncertainty of how to perform their role as objective decision-maker in relation to the emotional content of the victims’ narrative. Criminal justice professionals may feel like they have to move “outside” their professional objectivity to do accommodate the victim (Rudolfsson, 2022). Like in many other countries, many legal scholars in the Netherlands have drawn attention to the VIS’s potential to disrupt magistrates’ objectivity, especially when the scope of the VIS was extended in 2016. As the number of VISs that are presented are relatively low in comparison to the total number of cases tried, many magistrates have not yet got the opportunity to get fully accustomed to the practice of the VIS. Laws and guidelines are characterized by a rather flexible approach (Booth et al., 2018; Lens et al., 2010), leaving magistrates high levels of discretion on how to manage the courtroom proceedings.

There is yet another interpretation of being in two minds. The current study shows that magistrates make an effort to empathize with both the defendant *and* the victim. In the literature, the courtroom is sometimes described as a “three-team interplay” (Flower, 2018; Goffman, 1956), referring to the judge, the prosecutor and the defendant. The victim, who is not a party to the criminal proceedings in Dutch law, but does participate in the proceedings, presents a new three-team. Up until the designated moment for the VIS, there is little attention to the victim in the room. However, that changes as soon as the judge announces the VIS. Judges will try to create a safe moment isolated from the rest of the hearing in which the victim may speak uninterruptedly: the three-team of judge, prosecutor and victim.

After the victim finishes presenting the VIS, the judge opens up a space in which empathy may be established. Not only between victim and magistrate, but also between victim and defendant. The defendant does not communicate directly with the victim, but via the presiding judge. This gives the judge a position in which emotional labor is at its peak: the judge creates room for empathy, but has to guard for negative reactions. After the closing of the VIS, the presence of the victim and the message of the VIS lingers. The judge often uses it to create an atmosphere in which the defendant gets more talkative. The verdict does not always explicitly address the statement, but if the victim is present during the delivery of the VIS, there is another opportunity for the judge to empathize with the victim.

Overall, the study confirms that judges empathize with everyone rather than with no one. The distinction between empathy and sympathy is useful to see that this empathizing does not threaten the magistrates’ objectivity. If magistrates would sympathize—placing the needs and perspectives of one party categorically superior to another’s—that would be problematic. However, there were multiple examples in this paper where the judge or prosecutor showed equal regard for all parties perspectives.

Concluding, this paper shows that judges accommodate the victims’ voice in Dutch criminal law, while succeeding in remaining objective decision-makers. However, it should be noted that the sample of observations is in a sense a very skewed sample: most victims do not reach the point of delivering a VIS in court. Apart from the justice gap due to low attrition rates—especially in cases of sexual assault—even victims whose case is tried, many choose not to present an oral VIS or do not get the opportunity to do so, either because of eligibility or because something went wrong in the preparation phase. Because the oral VIS seems to be a turning point in the trial for including the victims’ perspective, it remains to be seen to what extent their voice is accommodated for the written VIS and to what extent victim acknowledgment is achieved if there is no VIS delivered.

## Data Availability

The data analyzed in this study is subject to the following licenses/restrictions: The dataset is owned by another organization (WODC, The Netherlands). Permission was granted to analyse the data for this purpose, but the dataset cannot be shared. Requests to access these datasets should be directed to abosma@nscr.nl.

## Footnotes

^1^The potential of healing should be regarded with care as the potential for closure is easily overstated – which may be harmful in itself, see Bandes (2022).

^2^There are currently no reliable statistics about the number of VISs in Dutch criminal procedure, because of inconsistent registration, but it is clear that cases with oral VISs presented are the exception to the rule.

^3^*If applicable, especially witness and expert interrogation is quite uncommon, as evidence is gathered in pre-trial investigations.

^4^Other noteworthy amendments relate to the number of surviving family members who are allowed to present an oral VIS during trial (increased from 1 to 3 in 2012) and the 2021 law that introduced (1) the inclusion of stepfamily as representatives of the victim, (2) the requirement for the defendant to be present during a trial in which a victim presents a VIS and the fixed moment for the VIS during trial.

^5^Victim Support the Netherlands notified researchers about a case in which the victim intended to present an oral VIS.

^6^The case in which 40 years passed between the date of the crime and the date of the hearing is a case about war crimes committed abroad. Another case in which a notable amount of time passed between the date of the crime and the date of the hearing, namely 25 years, was a case about sexual assault of minors.

^7^No notable differences were found between districts. The Netherlands is a relatively small country. Although the judiciary is divided in 10 districts, they are governed by national laws and policies. Practice may differ slightly, but no notable differences were found in the observations.

^8^Related to the MH-17 case. Flight MH17, departing from Amsterdam heading towards Kuala Lumpur, crashed on July 17, 2014 in the Ukraine due to a missile impact. All 298 people on board were killed. 91 surviving family members presented an oral statement during trial (www.courtmh17.com). For more information on victim participation in this case, see Buiter et al. (2022).

^9^They may react form-free, but most victims who actively engage with the criminal proceedings make their wishes known using this form.

## References

[ref1] Bandes S. A. (2009). Empathetic judging and the rule of law. Cardozo Law Rev. De Novo 133, 133–148.

[ref2] Bandes S. A. (2022). What are victim impact statements for? Brooklyn Law Rev. 87, 1253–1282.

[ref3] Bandes S. A. Lynée Madeira J. Temple K. Kidd White E. (2021). Research handbook on law and emotion. Cheltenham: Edward Elgar.

[ref4] Bergman Blix S. Wettergren Å. (2018). The emotional interaction of judicial objectivity. Onati Socio-Legal Ser. 9, 726–746. doi: 10.35295/osls.iisl/0000-0000-0000-1031

[ref5] Bernstein J. M. (1991). “Grand narratives” in On Paul Ricoeur: narrative and interpretation. ed. Wood D. (London: Routledge), 102–123.

[ref6] Bibas S. Bierschbach R. A. (2004). Integrating remorse and apology into criminal procedure. Yale Law J. 114, 85–148. doi: 10.2307/4135717

[ref7] Booth T. (2012). 'Cooling out' victims of crime: managing victim participation in the sentencing process in a superior sentencing court. Aust. N. Z. J. Criminol. 45, 214–230. doi: 10.1177/0004865812443680

[ref8] Booth T. Bosma A. K. Lens K. M. E. (2018). Accommodating the expressive function of victim impact statements: the scope for victims’ voices in Dutch courtrooms. Br. J. Criminol. 58, 1480–1498. doi: 10.1093/bjc/azy001

[ref9] Bosma A. K. (2019). Emotive justice. Laypersons’ and legal professionals’ evaluation of emotional victims within the just world paradigm. Tilburg: Wolf Legal Publishers.

[ref10] Bosma A. K. Groenhuijsen M. S. de Vries M. (2021). Victims’ participation rights in the post-sentencing phase: the Netherlands in comparative perspective. N. J. Eur. Crim. Law 12, 128–145. doi: 10.1177/20322844211008232

[ref11] Buiter M. Y. Boelen P. A. Kunst M. Gerlsma C. de Keijser J. W. Lenferink L. I. M. (2022). The mediating role of state anger in de associations between intentions to participate in the criminal trial and psychopathology in traumatically bereaved people. Int. J. Law Psychiatry 85:101840. doi: 10.1016/j.ijlp.2022.101840, PMID: 36274496

[ref12] Dijkstra S. (2017). De pratende, schrijvende en twitterende rechter: Terughoudendheid troef. Rechtstreeks. 1, 13–28.

[ref13] Field S. Tata C. (2023). Criminal justice and the ideal defendant in the making of remorse and responsibility. Oxford: Hart Publishing.

[ref14] Flower L. (2018). Loyalty work: emotional interactions of defence lawyers in Swedish courtrooms. Lund: Lund University.

[ref15] Goffman E. (1956). The presentation of self in everyday life. Edinburgh: University of Edinburgh Social Science Research Centre.

[ref16] Goodrum S. (2013). Bridging the gap between prosecutors’ cases and victims’ biographies in the criminal justice system through shared emotions. Law Soc. Inq. 38, 257–287. doi: 10.1111/lsi.12020

[ref17] Haket V. (2007). Veranderende verhalen in het strafrecht. De ontwikkeling van verhalen over verkrachting in het strafproces. Ridderkerk: Ridderprint.

[ref18] Hochschild A. R. (2003). The managed heart: commercialization of human feeling. 2nd *Edn.* Berkeley: University of California Press.

[ref19] Johansen L. V. Adrian L. Asmussen I. H. Holmberg L. (2023). The power of professional ideals: understanding and handling victims’ emotions in criminal cases. Int. Rev. Vict. 29, 236–258. doi: 10.1177/02697580221100566

[ref20] Karstedt S. (2011). “Handle with care: emotions, crime and justice” in Emotions, crime and justice. eds. Karstedt S. Loader I. Strang H. (Oxford: Hart Publishing), 1–19.

[ref21] Kragting M. Augusteijn F. Elbers N. A. de Waardt M. Beijers J. Pemberton A.. (2022). Evaluatie wet uitbreiding spreekrecht slachtoffers en nabestaanden in het strafproces. Amsterdam: NSCR/Universiteit Leiden.

[ref22] Kragting M. Elbers N. A. Augusteijn F. de Waardt M. Beijers J. Kunst M.. (2024). Understanding the relation between agency and communion and victim impact statements. Int. Criminol. 4, 66–78. doi: 10.1007/s43576-024-00116-6

[ref23] Lens K. M. E. Pemberton A. Groenhuijsen M. S. (2010). Het spreekrecht in Nederland: Een bijdrage aan het emotioneel herstel van slachtoffers? Tilburg: INTERVICT.

[ref24] Mäkelä M. Björninen S. (2022). “My story, your narrative” in The Routledge companion to narrative theory. New York: Routledge, 11–23.

[ref25] Maroney T. A. (2011). The persistent cultural script of judicial dispassion. Calif. Law Rev. 99, 629–682. doi: 10.15779/Z38K98M

[ref26] Milka A. Lemmings D. (2017). Narratives of feeling and majesty: mediated emotions in the eighteenth-century criminal courtrom. J. Legal History 38, 155–178. doi: 10.1080/01440365.2017.1336891

[ref27] Pemberton A. Bosma A. K. (2024). Legal fictions in various forms of victim participation. Int. Criminol. 4, 55–65. doi: 10.1007/s43576-024-00115-7

[ref28] Roach Anleu S. Mack K. (2005). Magistrates' everyday work and emotional labour. J. Law Soc. 32, 590–614. doi: 10.1111/j.1467-6478.2005.00339.x

[ref29] Rogers L. J. Erez E. (1999). The contextuality of objectivity in sentencing among legal professionals in South Australia. Int. J. Sociol. Law 27, 267–286. doi: 10.1006/ijsl.1999.0092

[ref30] Rudolfsson L. (2022). “At least I tried”: Swedish police officers’ experiences of meeting with women who were raped. J. Police Crim. Psychol. 37, 365–376. doi: 10.1007/s11896-021-09435-0

[ref31] Ryan A. (2023). The form of forms: everyday enablers of access to justice. Soc. Leg. Stud. 32, 690–713. doi: 10.1177/09646639231172616, PMID: 30923868

[ref32] Schnädelbach S. (2018). Engineerd emotions: Court rhetoric and feeling rules from the German Kaiserreich to the Weiman Republic Workshop judging, emotion and emotion work. Spain: IISJ Oñati.

[ref33] Shklar J. (1964). Legalism: law, morals and political trials. Cambridge: Harvard University Press.

[ref34] Shklar J. (1990). The faces of injustice. New Haven: Yale University Press.

[ref35] Shuler S. Sypher B. D. (2000). Seeking emotional labor: when managing the heart enhances the work experience. Manag. Commun. Q. 14, 50–89. doi: 10.1177/0893318900141003

[ref36] Tata C. (1997). Conceptions and representations of the sentencing decision process. J Law Soc 24, 395–420. doi: 10.1111/j.1467-6478.1997.tb00004.x

[ref37] Törnqvist N. (2022). Drizzling sympathy: ideal victims and flows of sympathy in Swedish courts. Int. Rev. Victimol. 28, 263–285. doi: 10.1177/02697580211035586

[ref38] van Oorschot I. Mascini P. Weenink D. (2017). Remorse in context(s): a qualitative exploration of the negotiation of remorse and its consequences. Soc. Leg. Stud. 26, 359–377. doi: 10.1177/0964663916679039

